# Immunohistochemical localization patterns for vimentin and other intermediate filaments in calcified ovarian fibrothecoma

**DOI:** 10.1186/1746-1596-1-28

**Published:** 2006-09-11

**Authors:** Eric Scott Sills, Terrence B Doan, R James Mock, George R Dixson, Michael B Rohlfing

**Affiliations:** 1 Department of Obstetrics, Gynecology & Reproductive Research, Murphy Medical Center, Murphy, NC, USA; 2 Department of Surgery, Murphy Medical Center, Murphy, NC, USA; 3 Gastroenterology Division, Department of Medicine, Murphy Medical Center, Murphy, NC, USA; 4 Department of Radiology, Murphy Medical Center, Murphy, NC, USA; 5 Department of Pathology, Murphy Medical Center, Murphy, NC, USA; 6 75 Medical Park, Suite D, Murphy, NC 28906, USA

## Abstract

**Problem:**

To describe immunohistochemical features encountered in ovarian fibrothecoma with correlation to clinical presentation and surgical management.

**Method of study:**

A female age 75 presented for evaluation of melena. The patient reported total abdominal hysterectomy and removal of both ovaries 40 years earlier.

**Results:**

CA-125 was normal and there was no evidence of hyperestrogen effect. Pelvic CT revealed a partially calcified 7 cm pelvic mass without adenopathy or ascites; ultrasound was confirmatory. Endoscopy identified three benign intestinal tubular adenomas. Following laparoscopic excision of the pelvic tumor immunohistochemichal analysis of the mass showed negative staining for keratin, S100 protein, inhibin, calretinin, melan A, smooth muscle actin, CD34, CD117, and desmin. The tissue was positive for vimentin, however.

**Conclusion:**

Ovarian fibrothecomas represent an ovarian stromal neoplasm developing in a wide spectrum of clinical settings. Particularly if oophorectomy is stated to have been performed remote from the time of index presentation, the status of the ovaries must be considered whenever pelvic pathology is encountered. We describe a calcified ovarian fibrothecoma identified during gastroenterology investigation and confirmed immunohistochemically via high amplitude vimentin signal.

## Background

Stromal tumors of the ovary include thecoma and fibroma, yet as differentiation between these two types may be difficult the term fibrothecoma has emerged in recognition of the similar immunohistochemical features present in both. The exact incidence of fibrothecoma is unknown, although they have been described as rare ovarian neoplasms [1]. Here we present an unusual clinical manifestation of calcified ovarian fibrothecoma in the absence of ascites, arising from a residual ovary intentionally conserved at laparotomy 40 years earlier.

## Case report

A non-smoking 75 year old Caucasian female presented for gastroenterology evaluation due to rectal bleeding. She had mild essential hypertension well controlled on diltiazem, did not smoke, and had no other medical complaint. She reported intermittent oral use of aspirin, 81 mg/d. In 1966, she underwent total abdominal hysterectomy "when both ovaries were removed". At the time of our evaluation, the patient used supplemental estrogen occasionally but was uncertain when this commenced. Her only other surgery was an uncomplicated laparoscopic cholecystectomy performed in 1996.

**Figure 1:**
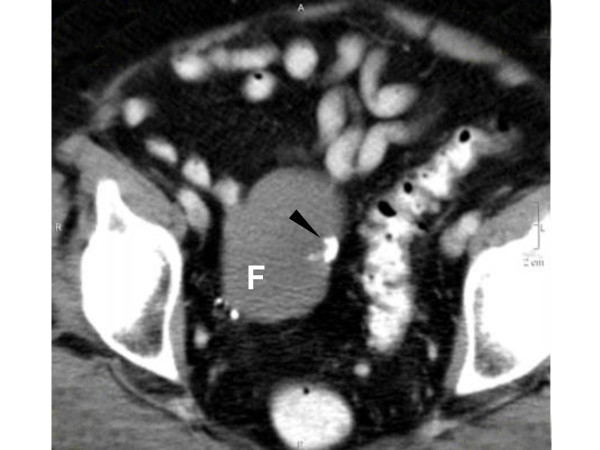
Pelvic CT demonstrating 7 cm fibrothecoma (F) in the upper right pelvis and associated calcification (arrow). No ascites or bowel dilation is present. Uterus and left ovary are surgically absent in this 75 year-old patient.

The patient's BMI was 26. Vital signs and physical exam were normal although a heme positive stool was noted. Abdominal CT showed no significant abnormality, although pelvic CT revealed a 7 cm partially calcified mass at the upper pelvic inlet, indistinguishable from bowel [Figure 1]. Ultrasound of this lesion confirmed a 74 × 45 × 49 mm heterogeneous (partially echogenic) mass in the upper right pelvis.

Serum electrolytes were unremarkable except for BUN and Cr at 20 mg/dl and 1.1 mg/dl, respectively. Hemoglobin was 11.8 g/dl. Serum hCG, CEA, and CA-125 were all normal.

### Surgical management

**Figure 2:**
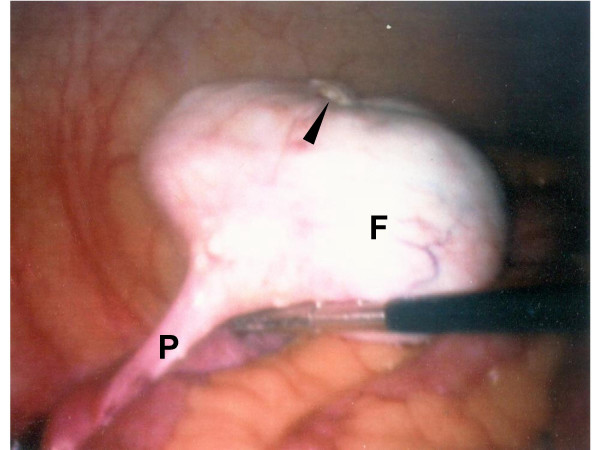
Right ovarian fibrothecoma (F) and pedicle (P) at laparoscopy. Calcified nodule is shown superiorly (arrow).

A 12 mm umbilical port was placed along with two accessory ports (both 5 mm caliber), one each at the left upper and lower quadrants. Extensive abdominal adhesions were encountered and lysed with blunt dissection and harmonic scalpel. Eventually an approximately 7 cm smooth, white glistening mass could be seen in the right aspect of the pelvis [Figure 2]. The mass was attached laterally to the right pelvic sidewall; these connections were secured with 10 mm endosurgical clips and then divided to free the tumor. Survey of abdomen and pelvis revealed no other gross abnormality. Estimated blood loss was ~50 ml and the patient was discharged home the same afternoon in excellent condition.

### Immunohistochemistry protocol

**Figure 3:**
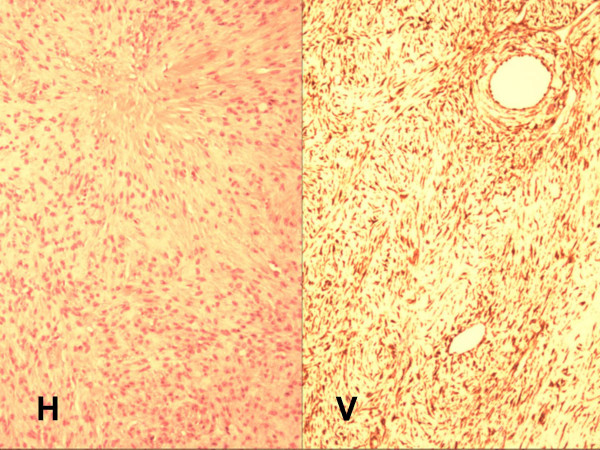
Microscopic features of ovarian fibrothecoma include round and spindle shaped nuclei without atypia or myxoid change. No mitotic figures were observed. Standard H&E (H) and vimentin stain (V), 40× magnification.

Formalin-fixed tumor sections embedded in paraffin were exposed to 10 mmol/L citrate buffer (pH 6.0), followed by incubation with 1:50 dilution of monoclonal mouse anti-vimentin (M0725, DakoCytomation, Carpinteria, CA, USA) using 20 min heat-induced epitope retrieval in DakoCytomation Target Retrieval solution (S3308) × 30 min incubation at room temperature with primary antibody. Negative control was 1:50 dilution mouse IgG1 (X0931) which was run simultaneously. Complexes were visualized via DAKO LSAB+/HRP kit (K0679) and automated stainer platform. The specimen demonstrated positive vimentin staining for mesenchymal spindle and round cells, consistent with benign ovarian stromal neoplasm [Figure 3]. The full panel consisted of antibodies to keratin, S100, inhibin, calretinin, melanoma associated marker "Melan A", smooth muscle actin, CD34, CD117 and desmin, but none returned a positive result [Table 1].

**Table 1:** Immunohistochemical characteristics observed in calcified ovarian fibrothecoma without ascites.

**Assay/antibody**	**Tissue marker for**	**Result**
Vimentin	Vimentin, intermediate filaments, mesenchymal cells	**+**
CAM 5.2	Keratin: 39, 43, 48, 50, 50.6 kD	**-**
S100	S100 Protein, nerve sheath tumor, melanoma, chondrocyte	**-**
Inhibin	Sex cord stromal tumors, adrenal (cortical) tumors	**-**
Calretinin	Ca^++ ^binding protein, mesothelial cells, sex cord stromal tumors	**-**
Melan A	Melanoma associated marker, adrenal (cortical) tumors	**-**
SMA	Smooth muscle actin, myofibroblasts, myoepithelial cells	**-**
CD34	Endothelial and stem cells, GI stromal tumor	**-**
CD117	Myeloid and mast cells, GI stromal tumor (c-kit)	**-**
Desmin	Muscle, desmoplastic small round cell tumor	**-**

## Discussion

We report an unusual case of partially calcified ovarian fibrothecoma without ascites in an elderly female reporting previous oophorectomy and complaining of melena. For this patient, her gastrointestinal bleeding was determined to be secondary to benign polyps, although it was during the work-up associated with this lesion that suspicion was raised regarding a 7 cm calcified right pelvic mass. While this mass did appear benign intraoperatively, metal endosurgical clips were deployed to assist subsequent radiographic localization of tumor site in the event adjuvant radiotherapy was indicated. Excision of the tumor at laparoscopy confirmed its ovarian origin, and immunohistochemical labeling was performed to characterize it more fully as a benign fibrothecoma.

Other investigators have commented on the protean nature of ovarian tumors in this group that may occur in the setting of edema [2], elevated CA-125 [3,4], and pregnancy [5]. While immunohistochemical features of this neoplasm have been described previously [6-8], the thecoma-fibroma group of ovarian stromal tumors represents a spectrum of lesions in which clear distinctions between various entities are difficult to define^6^. However, tumor calcification and low serum CA-125 are infrequent findings.

One finding that proved useful in finalizing the diagnosis for our patient was the high affinity for vimentin staining, characteristic of ovarian fibrothecoma [2]. Vimentin is a 57 kD intermediate filament protein forming the cytoskeleton of vertebrate cells. The protein was initially thought to be preferentially retained in malignant mesenchymal tissues, although coexpression of intermediate filaments (particularly cytokeratin and vimentin) was subsequently shown to exist in many benign lesions. Accordingly, application of multiple antibodies is recommended to formulate a diagnosis with sufficient precision, as demonstrated in our case. Indeed for this calcified ovarian fibrothecoma, vimentin was the only positive immunohistochemical marker registered from a panel of ten antibodies.

The impact of patient recall error while obtaining the surgical history also warrants comment. A variant of residual ovary syndrome, the problem begins when the patient is unaware of an ovary intentionally conserved at surgery. The ovary subsequently develops a pathologic process or causes symptoms necessitating its removal in a second operation [9]. Not surprisingly, this is more likely among subjects of advanced age who may have never known (or forgotten) important details of an operation performed long ago and for which no written record can be obtained. Our patient had believed that she had no ovaries for 40 years, a "fact" dutifully reported to multiple physicians over time. Could awareness of her retained single ovary have alerted this patient's caregivers to the presence of ovarian fibrothecoma sooner? Certainly when hysterectomy-oophorectomy is stated to have been performed remote from the time of index presentation, an *in situ *ovary should still be considered whenever pelvic pathology is encountered.
